# Strategies and foundations for scientific discovery in longitudinal studies of bipolar disorder

**DOI:** 10.1111/bdi.13198

**Published:** 2022-03-18

**Authors:** Melvin G. McInnis, Ole A. Andreassen, Ana C. Andreazza, Uri Alon, Michael Berk, Teri Brister, Katherine E. Burdick, Donghong Cui, Mark Frye, Marion Leboyer, Philip B. Mitchell, Kathleen Merikangas, Andrew A. Nierenberg, John I. Nurnberger, Daniel Pham, Eduard Vieta, Lakshmi N. Yatham, Allan H. Young

**Affiliations:** ^1^ Department of Psychiatry University of Michigan Ann Arbor Michigan USA; ^2^ NORMENT Centre University of Oslo and Oslo University Hospital Oslo Norway; ^3^ Department of Pharmacology & Toxicology Temerty Faculty of Medicine University of Toronto Toronto Ontario Canada; ^4^ Weizmann Institute Rehovot Israel; ^5^ Deakin University IMPACT – the Institute for Mental and Physical Health and Clinical Translation School of Medicine Barwon Health Geelong Australia; ^6^ Orygen The National Centre of Excellence in Youth Mental Health Centre for Youth Mental Health Florey Institute for Neuroscience and Mental Health and the Department of Psychiatry The University of Melbourne Melbourne Australia; ^7^ National Alliance on Mental Illness Arlington Virginia USA; ^8^ Brigham Womens Hospital Harvard University Boston Massachusetts USA; ^9^ Shanghai Mental Health Center Shanghai Jiao Tong University School of Medicine Shanghai Mental Health Center Shangai China; ^10^ Mayo Clinic Rochester Minnesota USA; ^11^ Département de psychiatrie Université Paris Est Creteil (UPEC) AP‐HP Hôpitaux Universitaires H. Mondor DMU IMPACT INSERM, translational Neuropsychiatry Fondation FondaMental Creteil France; ^12^ University of New South Wales Sydney Australia; ^13^ Intramural Research Program National Institute of Mental Health Bethesda Maryland USA; ^14^ Mass General Hospital Harvard University Boston Massachusetts USA; ^15^ Indiana University Indianapolis Indiana USA; ^16^ Milken Institute Center for Strategic Philanthopy Washington District of Columbia USA; ^17^ Bipolar and Depressive disorders Unit Hospital Clinic Institute of Neuroscience University of Barcelona IDIBAPS CIBERSAM Barcelona Catalonia Spain; ^18^ University of British Columbia Vancouver British Columbia Canada; ^19^ Department of Psychological Medicine Institute of Psychiatry, Psychology and Neuroscience King’s College London & South London and Maudsley NHS Foundation Trust Bethlem Royal Hospital Beckenham Kent UK

**Keywords:** behavior, circadian, ontology, outcomes, personality, psychology

## Abstract

Bipolar disorder (BD) is a complex and dynamic condition with a typical onset in late adolescence or early adulthood followed by an episodic course with intervening periods of subthreshold symptoms or euthymia. It is complicated by the accumulation of comorbid medical and psychiatric disorders. The etiology of BD remains unknown and no reliable biological markers have yet been identified. This is likely due to lack of comprehensive ontological framework and, most importantly, the fact that most studies have been based on small nonrepresentative clinical samples with cross‐sectional designs. We propose to establish large, global longitudinal cohorts of BD studied consistently in a multidimensional and multidisciplinary manner to determine etiology and help improve treatment. Herein we propose collection of a broad range of data that reflect the heterogenic phenotypic manifestations of BD that include dimensional and categorical measures of mood, neurocognitive, personality, behavior, sleep and circadian, life‐story, and outcomes domains. In combination with genetic and biological information such an approach promotes the integrating and harmonizing of data within and across current ontology systems while supporting a paradigm shift that will facilitate discovery and become the basis for novel hypotheses.

Bipolar disorder (BD) is a heterogeneous common condition, affecting 4.4% of the population when subthreshold manifestations are included.^1^ It is characterized by cyclying between periods of relative wellness and variable degrees of illness severity (disease states) that range from the extremes of mania or depression to chronic persistent low‐grade abnormalities of mood.^2^ BD is highly heritable with a complex pleiotropic polygenetic background.^3^ The unifying feature of the illness is the dynamic variability of energy and activity over time, with clinical manifestations that are both psychological (mood, affect, and cognition)^4^ and physical (motor and visceral).^5^ Although the efficacy of combined medication and adjunctive psychotherapy^6^, ^7^ has been established, the consequences of BD in terms of impairment in life roles, comorbid substance abuse, and medical conditions highlight the importance of early detection and prospective tracking across the life span.

The discovery of novel treatments will depend on a rational and systematic approach, targeting the mood instability that is a primary feature of BD. It is noteworthy that medications discovered for other purposes are secondarily adapted for use in BD.^8^, ^9^ The modest and similar effect size of the recent (past 20 years) medication offerings from the second generation antipsychotic class provides a compelling argument for the need for personalized and novel treatments designed specifically for BD.^10^ The process must focus on rigorous science, yet allowing and even encouraging fortuitous discovery such as was the case for lithium.^11^ Such an approach demands an ontological framework and intellectual infrastructure around which to collect and order outcomes data from cohorts designed to capture clinical, cultural, and geographical diversity.^12^ A dedicated team of multidisciplinary researchers with expertise across the clinical, cultural, and basic science domains must be engaged and empowered toward discovery.^13^ Finally, a sustainable financial support model is necessary to ensure that researchers and participants alike are kept active, respected, and engaged in a partnership in the active expansion of knowledge.^12^ Resources for BD have historically lagged behind other illnesses and efforts have further declined in recent years.^14^

We believe that a significant paradigm shift as defined by Thomas Kuhn in his influential *Structure of Scientific Revolutions* ^15^ is needed to rapidly advance knowledge in understanding and to aid the discovery of treatments for BD. Kuhn points to four fundamental elements of a paradigm: ontology (what is), epistemology (how is it known), methodology (how to), and axiology (value of). The ontological platform is the focal point upon which to collect, manage, and analyze information and knowledge gained over time; it facilitates mapping or linking the clinical data with the basic sciences and harmonizing across large‐scale longitudinal cohorts.^16^, ^17^ Lacking a central falsifiable hypothesis behind the essence of the disorder, the field must begin with an atheoretical signal discovery process beginning and anchored in well characterized, yet diverse, cohorts.

We begin with a discussion of ontology as the base to support the global initiatives and resources needed to create a diverse, multidisciplinary, and multidimensional endeavor that integrates clinical and biological data while embracing the priniciples of open science.^18^, ^19^ Our approach adapts current ontological systems that recognize modularity inherent in biological systems.^20^ Biological systems naturally and efficiently aggregate in modules. The ontological platforms outlined herein are modular systems that naturally interconnect and integrate across platforms with resulting consequences at the clinical level. The emerging paradigm we set forth provides the basis for the next generation research in BD, one that develops deep knowledge of the ontological modules, discovers how these modules interact, and the trajectory of events that lead to medical and psychiatric consequences that are the observed phenotypes that we recognize as BD.

## 1 Clinical and phenomenological basis for BD

BD is a complex, dynamic, and heterogeneous condition with varied psychopathology.^21^, ^22^, ^23^, ^24^ It is commonly associated with multiple psychiatric and somatic comorbidities, and poorly understood pathophysiology.^25^, ^26^ There are many unconfirmed biochemical abnormalities hypothesized in the pathophysiology of the disorder.^27^ Further, the fundamental underlying *causes* of BD are far from being established, notwithstanding a multitude of suggested contributing factors.^28^ Discrete illness states (categories) – mania, depression, and hypomania – are clinically defined by combinations of signs and symptoms in the current Diagnostic and Stastical Manual 5 (DSM 5).^26^ These categories are *disjunctive*, a sign or symptom may belong to multiple DSM categories (e.g. sleep disturbance or irritability can be part of both mania and depression), and *discordant* (the same person may experience different elements within the category from one episode to the next). Furthermore, category boundaries are blurred and confounded by variable intensity of symptoms and fluctuating overlap of depressive and manic symptoms (mixed states).^29^ Comorbidity with other psychiatric psychopathology such as personality disorder, and attention deficit hyperactivity disorder; substance abuse is common and complicates diagnosis while adding to the overall disability.^30^ The inter‐episodic periods are often productive and enjoyed in good health, yet may include multiple medical comorbidities,^31^ ongoing or periodic substhreshold affective symptoms,^32^ and impaired cognitive functioning leading ultimately to chronic disability.^33^

The core pathognomonic state of BD is mania, a state of pathologically elevated energy and activity.^26^ Outside of rare instances of focal brain damage and stimulant or steroid‐induced states, there are few human conditions besides BD that manifest with mania.^34^ Many descriptions of the clinical states of BD have emerged over the past century, but none match the vivid and detailed text of Kraepelin in his treatise *Manic Depressive Insanity and Paranoia*, wherein he offers insight into the natural course of the disorder, unencumbered by effective treatments.^35^ These prescient observations form the basis for current classification systems of psychiatric illnesses.^26^, ^36^ They remain useful as clinical anchors, but it is recognized that much more, i.e. a fundamental paradigm change, is necessary to begin to unravel causality.^37^

We suggest that an ontological infrastructure or framework provides the base for such a paradigm shift to organize, link, and interpret multidisciplinary information.

## 2 Establishing an ontological base for bipolar disorder

Assembling an ontological framework and infrastructure for BD will include contributions from the clinical and basic sciences, as well as those with lived experience. For subsequent epistemological validity, it will no longer be acceptable to approach BD solely from a genetic, neuroimaging, psychological, or sociological basis; rather an integrated multidimensional approach is needed, one that is diverse, interactive, collaborative, and global.

Multiple ontologies already exist^38^ and many are proposed,^39^, ^40^ their common elements being the annotation and integration of data with subsequent analyses leading ultimately to new knowledge.^16^, ^17^ Ontological systems offer a functional infrastructure for networking across multi‐modal / multidimensional / multidisciplinary entities in complex fields, such as mental health.^41^ A familiar example is the Gene Ontology (GO)^42^ system, it networks with clinical phenotype ontologies such as Disease Ontology (DO)^43^ or the DSM systems and offers pathways or sets of genes implicated in specific disorders. Integrated networks are key as reliance on single ontological systems (modules) has failed. The accurate and timely diagnosis of BD will depend on a range of disciplinary inputs within and across ontological systems; reliance on single systems such as the clinical presentation of mania, the current pathognomic feature of BD, results in significant delays in diagnosis.^44^

## 3 Toward an ontology for BD

**FIGURE 1:**
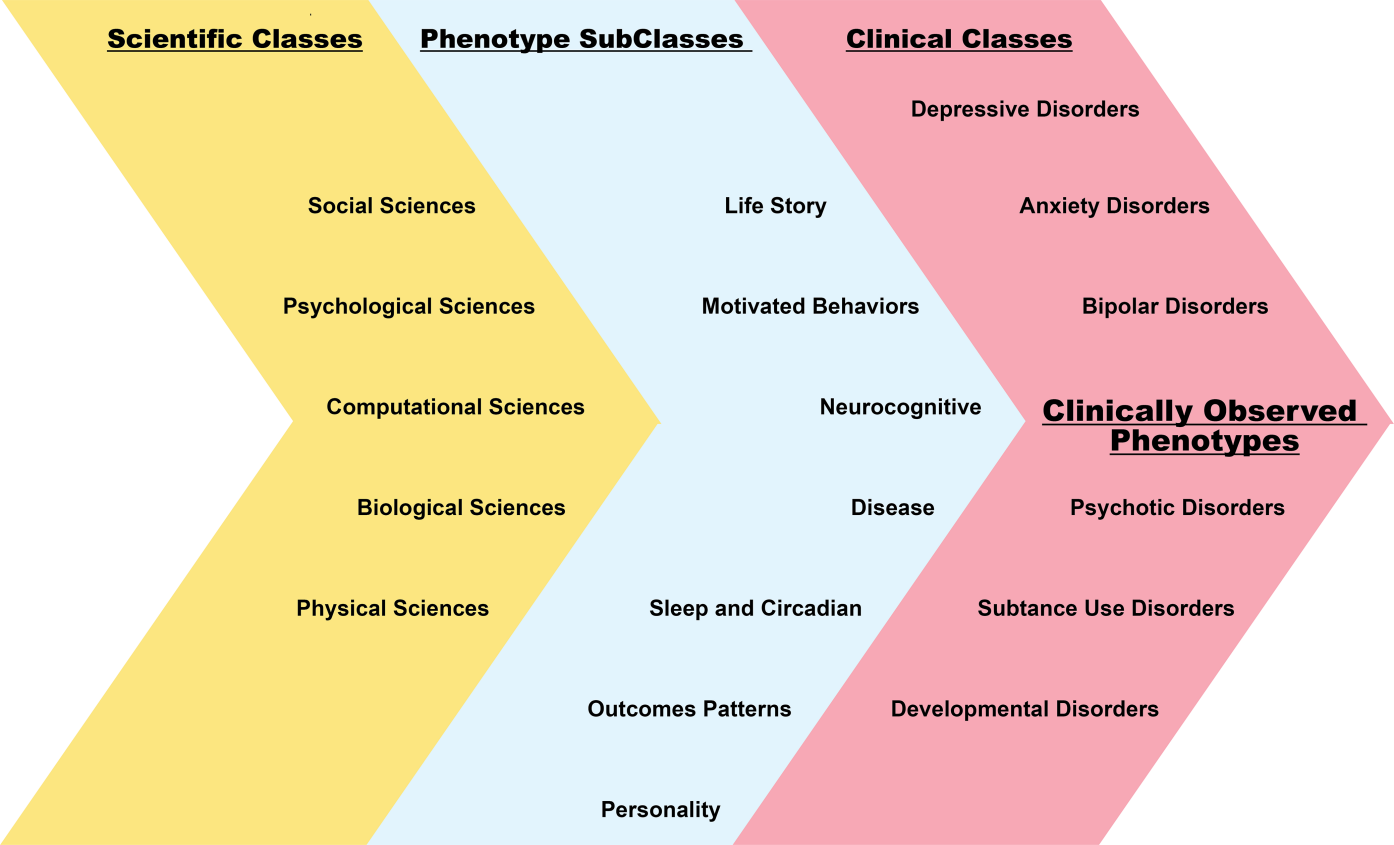
Clinically observed phenotypes include the disorders currently described in the standard categorical ontological systems such as the DSM and ICD. These phenotypes are the products of the contributions from a series of phenotypic subclasses that contribute to the observed clinical conditions in a manner that is variable in degree and intensity over time. The phenotypic subclasses are, in turn, the products of fundamental elements derived from the scientific classes (disciplines). For example in the biological sciences, genetics contributes to many if not most of the phenotypic subclasses that underlie the expression of mood disorders

An ontological system for BD builds on existing models,^38^, ^45^ essentially integrating ontologies from medical, biological, and social sciences. This structure has been implemented in the Prechter Bipolar Program at the University of Michigan,^46^ phenotypic subclasses are proposed that underlie and contribute to the observed phenomenological (clinical) phenotype (Figure 1).

## 4 Phenotype subclasses as ontological bases

### 4.1 Disease subclass

The concept of disease in psychiatric disorders is elusive, and mood disorders exemplify the blurred boundaries between pathological and nonpathological states in the human experience. Disease as a construct is notoriously difficult to define,^47^ and in the current classification systems (DSM and ICD) is a descriptive category, dependent on the clinical training, experience, and observation skills of the clinician.^26^, ^36^ BD is a clinically definable illness based on pathological expressions of affect,^48^ yet the boundaries of BD are obscured, for example by symptomatic nonspecificity, comorbidity, and the mixed affective states.^49^

BD as a *brain disease* is supported by genetic and biological observations.^28^ Neuroimaging‐based findings include evidence of structural and functional changes in the brain that support the disease construct.^50^ Biological mechanisms, biomarkers, and pathways have been implicated in BD, ranging from bioenergetics/mitochondria,^51^ the microbiome, circadian systems, and multiple *omics* and beyond.^52^ It has been hypothesised that these pathways converge on molecular bioenergetics and mitochondrial function which reflects the clinally biphasic bioenergetic nature of the disorder.^53^ Genetic studies identify risk loci,^3^ telomeres have been reported to be shortened,^54^ inflammatory and redox mechanisms are most likely involved,^55^ and there are indicators that BD has elements of a neurodevelopmental disorder.^28^

Progress in further discriminating subtypes within the BD category, e.g. BD I vs BD II, must ultimately be based on the underlying biology.^56^ There is evidence suggesting they may be distinct entities, with BD II mapping possibly closer to major depressive disorder than BD I, sharpening the need for a dynamic data framework to order and accommodate emerging data and expansions within existing ontologies.^39^, ^40^

### 4.2 Temperament and personality subclass

Temperament and personality have independent and competing ontologies by which to stratify this subclass. The first is categorical and includes a series of disjunctive sets of criteria that provide the basis for membership. This includes the DSM^26^ and ICD^36^ characterization of personality “disorders.” The challenges are exemplified by the fact that there are well over 100 combinations of symptoms that provide the clinician with the basis to make the diagnosis of borderline personality disorder, a disorder with diagnostic criteria that overlap with BD.^26^ This is a less than ideal manner to qualify a subclass.

The second approach to identifying strata within the temperament and personality subclass is partially dimensional and anchored in the descriptive *lexical hypothesis*. The origins are traced back to Francis Galton who appreciated the commonalities of mankind as revealed by language.^57^ This evolved to the description of five primary personality traits.^58^ These traits or “factors” are *neuroticism*, *extroversion*, *openness*, *conscientiousness*, and *agreeableness*, forming the *Big Five* traits of personality.^58^ These are measured in the clinical instrument NEO PI‐R^59^ and have been studied across a range of human conditions, including BD.^60^, ^61^

### 4.3 Neurocognitive subclass

Impairment in neurocognitive capacity is common in BD and may reflect, or be the result of, hypothesized underlying neural pathology in BD.^62^ Many psychiatric disorders manifest with impairments in neurocognitive abilities that raise the question of the relationship between the clinical disorder and the cognitive components.^63^ To what degree are the illness and cognitive elements a parallel process? Are they interdependent, i.e. does one cause the other? Is one a consequence of the other?

Impairment in several neurocognitive domains is common in BD, with disruptions in the domains of attention, memory, and executive functioning present in the euthymic state that are exacerbated with subthreshold mood symptoms.^64^ This is of significance as it is recognized that BD individuals are euthymic for less than half of the time in follow up, the remainder spent in varing degrees of syndromal or subsyndromal mood symptoms.^65^ This level of illness chronicity (residual mood symptoms) and cognitive impairment is consistently associated with poor levels of social, personal, and vocational functioning.^66^ Evidence is emerging for cognitive subgroups, endophenotypes defined within the neurocognitive sub‐phenotype class, of individuals within the BD diagnostic categories,^67^ and that in a subset of BD individuals these impairments are present at an early stage of the illness and may even precede the onset,^68^ while in others it may emerge as a result of neuroprogression.^69^

### 4.4 Motivated behaviors subclass

Motivated behaviors as an ontological subclass within psychiatry was proposed nearly 40 years ago by McHugh and Slavney.^70^ Motivation may be activated by internal or external stimuli and the resulting behaviors observed and measured at a personal level. As a compound term, *motivated behavior* combines complex constructs. *Motivation* implies a drive, providing and guiding the energy toward specific *behaviors* that represent sought out stimulus conditions appetizing to the individual, directed toward specific goals (e.g. eating, drinking, or sexual activity).^71^ Internal drives compel the behavior to consumption followed by a refractory period (e.g., after a satisfactory meal, hunger no longer drives food seeking behavior). Motivated behaviors are often according to the developmental stage of the individual; adolescent drives and motivations typically differ from those in adulthood and guide the study and understanding of social and developmental causation in human activity across the lifespan.^71^

In the study of BD, motivated behaviors are highly relevant and are linked to dysregulated bioenergic drivers. At a biochemical level, dopamine is arguably the critical motivational regulator and dopamine dysregulation lies at the heart of BD.^72^ Dopamine also is a key regulator of molecular bioenergetics. Internal states enhance or drive behaviors in specific circumstances and diminish them in others, typically in a highly personalized manner. Substance use disorders (SUD) are common among individuals with BD, with at least 50% experiencing SUD over their lifetime.^73^ The complexity of the relationship between BD and SUD is reflected in the relationship between mania, biological sex, and SUD; males with a higher occurence rate of mania are more likely to experience SUD.^74^ There are many overt behaviors that are driven in part by internal motivations, they include eating disorders, compulsive gambling, and other behaviors leading to self‐harm.^75^ While it can be argued that disease causes behavior, and behavior can cause disease, these concepts are clearly not interchangeable and should be evaluated independently, as well as in the context of associated phenomena.

### 4.5 Sleep and circadian patterns subclass

Individual sleep and circadian patterns have their own ontological structures that reflect both intrinsic rhythms and their interaction with environmental context,^76^ Findings from several studies converge in demonstrating a broad range of sleep disturbances, including variable sleep patterns, lower average and greater variability of motor activity, and a shift to later peak activity and sleep midpoint, indicative of greater evening orientation among people with BD.^77^ However, few studies have simultaneously considered these three domains simultaneously when characterizing rhythmic dysregulation in BD. The mismatch between *chronotype*, which refers to the nature of activity levels over the course of the day,^78^ and daily life schedules has been of particular interest in BD, and there is emerging data from high risk youth that shifts in the timing of rhythms occur during adolescence.^79^ These variable diurnal patterns have been proposed as endophenotypes in psychiatric disorders, and in particular BD.^80^ While a circadian ontology intersects with genetic ontologies, it is clear that there is much to be learned solely from the study of circadian patterns.^76^, ^81^ The circadian system is fundamental as it imposes structure to the physiology of the individual, providing a dynamic system that governs hormonal functions that vary over the course of the day and influences behaviors, interacting with the evolutionary drivers that likely originate in genetic pathways.^82^ Many of these pathways known to be dysregulated in BD, ranging from inflammation to oxidative biology and bioenergetic regulation, and are often under circadian control. This is reflected in the observation that several circadian genes and related functional variants have been repeatedly associated with BD.^81^

### 4.6 Life story as a phenotype subclass

The individual narrative of the person with a disease or condition is a fundamental component of medicine, the narrative history tells the story of context, exposures, and experiences in relation to disease.^83^ The ontological base for the life story phenotype subclass is in the measure of life events, behaviors, and the estimation of the impact on the personality, development, psychological schemas, vulnerability/resilience, health and well‐being of the individual.^84^ Several self‐report questionnaires have emerged to gather and quantify information on the personal history of life events.^85^ Life events and exposures are typically considered cumulative and add to stressors that increase the burden of disease; however, life events are not equal in impact and timing and while of significance, there are additional personal and dynamic factors that impact the influence of the life event at any given time.^86^ Further, the *expressed emotion*, measured by the number of critical comments in the personal environment and the family atmosphere of psychiatric patients, has been found to influence the early evolution of stressors and sustaining elements in BD.^87^ There is a very high rate of childhood trauma in BD, which contributes to risk for the illness.^88^ Social factors and inequities in the lives of individuals with BD also exert a variety of influences and deteriminants on outcomes. The advent of machine‐learnng approaches to extract profiles from text data (e.g. personal or medical records) will provide an important opportunity to harness the personal experiences of people with BD that may inform traditional assessment methods.^89^

### 4.7 Treatment response and outcome patterns, pharmacogenetics as a phenotype subclass

This phenotype subclass overlaps substantially with other subclasses and highlights the disjunctive nature of the elements within the subclasses. There is considerable variation in social, personal, and vocational functioning among those with BD, which contributes to the observed heterogeneity in outcomes of BD.^90^ Responses to medications vary substantially. The ontology behind pharmacogenetics is, to a significant degree, part of the gene ontology (GO) system, which provides an organization for individual genes according to type and function.^38^, ^42^ The GO system is, however, insufficient as response patterns are governed only in part by complex metabolism systems. Individuals are categorized as slow, intermediate, or fast metabolizers based on genotypes of a limited number of specific metabolizing enzymes.^91^ Medication response patterns may vary predictably (or unpredictably) according to combinations of many genetic variants across several metabolizing enzymes, and further complicated by medical comorbidities and concurrent medications.^28^ Finally, cultural influences and attitudes impact diagnosis and outcomes, specifically through the culturally specific expression of symptomatology and distress. This may affect diagnosis and adherence to medication management strategies.^92^

### 4.8 Embracing a paradigm shift – establishing ontological frameworks in longitudinal research

We are in the midst of a paradigmatic shift in our approach to BD research and care, it is driven by data and is a person‐centered process. The person centered path demands engagement of stakeholders in a dynamic and learning healthcare system aimed to improve outcomes at the individual and populations levels.^93^ Data drives the process and data demands order. The need for order requires an ontological framework: (1) to organize the exponentially increasing amounts of data that are generated in the research and clinical enterprise, (2) provide common platforms for related data types (modules) to be aggregated and whenever possible to be harmonized, (3) provide the base for coding data and data types in a consistent manner (harmonization), (4) facilitate links (integration) between data and data types that are reproducible and codified, and (5) facilitate the study of causality, the biological, psychological, and social consequences of the integral relationships between ontological modules or platforms.

A comprehensive approach to research and clinical care collects data and information from the elements described in Table 1. A comprehensive clinical assessment and formulation addresses these elements, but it is recognized that every patient in the clinical care setting will not undergo rigorous and detailed evaluation of each of the sub‐phenotypic classes, but clinicians form an impression on each subclass based on their clinical interview. In research setting, these elements are systematically evaluated and scored according to specific algorithms. How might research and clinical care be more integrated?

**TABLE 1:** The assessment of the person collects data under at least seven sub‐phenotypic classes. Each subclass is unique in describing characteristics of the person. The data types can be categorical, dimensional, or a combination of the two, i.e. a category can be further described in terms of intensity. Finally, the approach includes a series of clinical interviews, clinical lab assessments (such as in the neurocognitive sub‐phenotype class), and self‐report information

Sub‐Phenotype class	Description	Data types	Approach
Disease	What the person has	Category	Clinical interview assessment
Neurocognitive	How the person functions	Dimensional	Clinical lab assessment
Personality	Who the person is	Dimensional	Self‐report assessment
Life story	What happened to the person	Category/Dimensional	Self‐report assessment
Motivated behaviors	What the person does	Category/Dimensional	Clinical and self report assessment
Sleep and circadian	The daily rhythm of the person	Category/Dimensional	Clinical, lab, and self‐report assessment
Outcomes patterns	Trajectory of illness and treatment response of the person	Category/Dimensional	Clinical, lab, and self‐report assessment

Challenges and barriers in the progress in BD research are for the most part due to: (a) small sample sizes, (b) phenotype assessments limited to a focused interest, and (c) lack of comprehensive datasets that provide an indepth representation of the clinical and biological phenotypes.^28^, ^94^ A recent call to action has emphasized the importance of longitudinal studies involving large and comprehensive data collections based on a strategy of open science.^18^ Must we start completely afresh or can existing cohorts be used? How might we utlize existing samples throughout the world? Could the data be organized within ontological frameworks?

Large longitudinal cohorts, big data, genetics, integrated health records, phenomics, will be core drivers for expanding healthcare technology in the future.^95^ There are vast amounts of data already in the “system.” Recent data‐driven investigation involving information in the “system” pertaining to COVID‐19 revealed the weaknesses and deficits with the infrastructure to utilize very large and combined datasets.^96^ We are deep into a paradigm shift^15^ yet we find ourselves floundering. We submit that the floundering is at least in part secondary to the chaos in ordering the data. The critical ontological framework needed for order in BD should begin with the phenotypic subclasses outlined, creating and integrating ontological systems that are connected via a multidimensional matrix or network (Figure 1). Each of the phenotypic subclasses is derived from observations, assessments, or assays anchored in clinical or basic science disciplines, and each contributes to the clinically observed phenotype. The system is redundant; individual ontologies may contribute to multiple phenotypic subclasses, e.g. GO^42^ contributes to several phenotype subclasses. While the integration of ontological systems may be initiated with a supervised rules‐based approach,^97^ an iterative process is needed for dynamic relationships between modules (systems) to be evaluated and improved over time and potential axiomatic or causal relationships established in a given context.^98^

## 1 BD: A COMPLEX EPISODIC LIFETIME CONDITION AND THE NEED FOR LONGITUDINAL RESEARCH

Diverse and large longitudinal prospective cohorts specific to BD are necessary to discover a comprehensive understanding of the course and progression of the forms of BD. Detailed and personalized data are needed. While several broad and inclusive studies are emerging,^99^, ^100^, ^101^ few bring depth, detail, and focus needed to address the complexity of BD.^102^ Over the lifetime of an individual with BD, there are typically a series of acute and chronic states intermingled with periods of relative wellness and productivity. The patterns of interactions across the ontological modules over time are often complex, with the phenotypic subclasses contributing variably to the observed clinical states. Yet with sufficient detailed knowledge at the individual level, mathematical modeling of phenotypic patterns may provide a basis for stratification and prediction, and these strata become the basis for biological inquiry.^103^, ^104^, ^105^

## 2 PARADIGMS

Establishing prospective cohorts of BD are necessary but not sufficient to energize the paradigm shift. Universal and consistent strategies are needed to gather and organize phenotypic information electronically, either through available medical records, existing research data, or self‐report.^95^ Failure to implement the necessary ontological infrastructure to accommodate existing and emerging data will result in continued floundering, with data systems that are chronically insufficient and underdeveloped.^96^ The common *engine* of research, clinical care, education, learning health systems, or any paradigm that drives knowledge development is simply the *data*. The emerging paradigm proposed here recognizes the propensity for biological systems to aggregate (ontological platforms or modules), form interconnectiong networks, leading to causality.

## 3 CONCLUSION

The desire of the research community to conduct longitudinal studies in BD is surpassed only by the need. We begin by establishing worldwide cohorts and networks of BD individuals in the context of a well‐considered ontological and interconnected modular frameworks for research. This will provide a base for ongoing and future discovery‐oriented studies at the basic and clinical science levels.^18^, ^19^ Shared methods and protocols, as well as an open science approach, will ensure consistency and comparability across geographic regions. The ontological framework proposed herein is the necessary starting point and will be amended by emerging data and analyses over time. The knowledge gained will directly improve the lives of millions of people with BD, and as well provide fundamental insights into human mood and emotions.

## CONFLICT OF INTEREST

MGM has received consulting fees and research support from Janssen Pharmaceuticals. AAN received consulting fees, grants, or honoraria from Alkermes, Belvior Publishing, Ginger Inc., Merck, Myriad, Neuronetics, Patient Centered Outcomes Research Institute, Physician's Postgraduate Press, Protagenics, Slack Publishing, Sunovion, UpToDate Wolters Kluwer, and Wiley Publishing. JIN has received research support from Janssen Pharmaceuticals. MAF has financial interest in Chymia LLC and has received grant support from Assurex Health and the Mayo Foundation. LNY has been a speaker or a member of advisory board or received research grants from Alkermes, Abbvie, Allergan, Canadian Network for Mood and Anxiety Treatments, Canadian Institutes of Health Research, Sumitomo Dainippon Pharma, GlaxoSmithKline, Intracellular Therapies, Merck, Sanofi, and Sunovion, over the last 3 years. OAA is a consultant to HealthLytix, received speaker´s honorarium from Lundbeck and Sunovion. MB has received Grant/Research Support from the Wellcome Trust, Australian Research Council, Victorian Medical Research Accelerator Fund, Stanley Medical Research Foundation, National Health and Medical Research Council, Medical Research Futures Fund, Beyond Blue, A2 milk company, Meat and Livestock Board, Woolworths, Avant and the Harry Windsor Foundation, has been a consultant or speaker for Allergan, Eisai, Janssen and Janssen, Lundbeck, Merck, and Servier – all unrelated to this work. AHY has provided paid lectures and been a member on advisory boards for the following companies with drugs used in affective and related disorders: Astrazenaca, Eli Lilly, Lundbeck, Sunovion, Servier, Livanova, Janssen, Allegan, Bionomics, Sumitomo Dainippon Pharma, COMPASS, Sage, Novartis; all unrelated to this work. EV has received grants and served as consultant, advisor or CME speaker for the following entities (unrelated to the present work): AB‐Biotics, Abbvie, Aimentia, Angelini, Biogen, Celon, Dainippon Sumitomo Pharma, Ferrer, Gedeon Richter, GH Research, Glaxo Smith‐Kline, Janssen, Lundbeck, Organon, Otsuka, Sage, Sanofi‐Aventis, Sunovion, and Takeda

## Data Availability

There are no data relevant to this manuscript.
